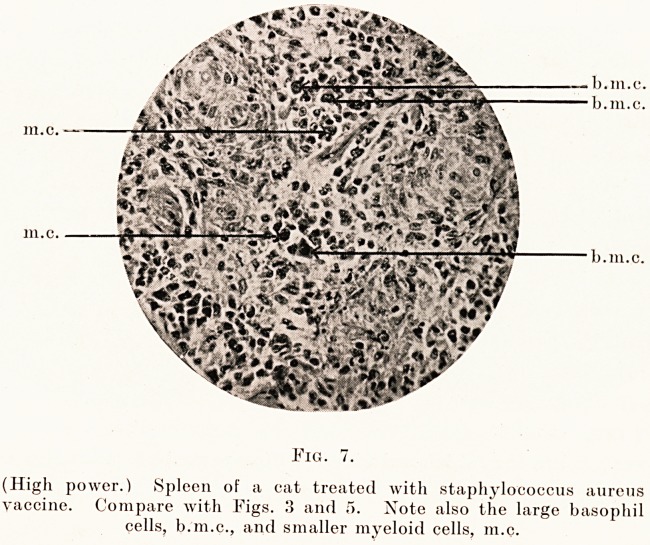# Changes in the Spleen in Acute Pyogenic Infections
*A thesis presented for the degree of M.D. in the University of Bristol.


**Published:** 1930

**Authors:** T. F. R. Hewer

**Affiliations:** Commonwealth Fund Fellow


### CHANGES IN THE SPLEEN IN ACUTE PYOGENIC INFECTIONS. *

BY

T. F. R. Hewer, M.D.,

*Commonwealth Fund Fellow.*

---

The enlargement of the spleen in acute infections has long been recognized by clinicians and pathologists alike. In typhoid fever, malaria and in all pyogenic infections one expects to find a palpable spleen. The typhoid spleen is seen at post-mortem to be an almost fluid mass of a deep red colour ; its increased size is due to the enormous number of red-blood corpuscles in the sinuses and in the pulp, and on section one sees many phagocytic cells, some of which are endothelial cells from the splenic venules, while others are macrophages similar to those found elsewhere : there are only a few nucleated cells in such a pulp, and if the hæmoglobin be washed out by fixation in alcohol the tissue looks rarefied under the microscope. Such changes as these are entirely different from those to be discussed in this paper, and the splenomegaly of protozoal diseases will not be considered.

In practically all acute pyogenic infections, but especially in septicæmias, the spleen becomes tumefied, so that it is readily palpable beneath the costal

* A thesis presented for the degree of M.D. in the University of Bristol.

margin. Its general appearance has been described by MacCallum,[1] and the following is taken from his account. Its size varies greatly, but its weight, in adults, may reach 600 or 700 grams or more. The capsule is tense, but the organ is soft, so that when it is cut the surface swells forward, everting the edges of the capsule. One may scrape off with the knife a quantity of smeary, paint-like pulp. The spleen may even be so soft that it flows as a semi-fluid material. The trabeculæ are sunken below the swollen surface, or else, if the cut surface has been scraped, they alone may be left as shaggy threads after the pulp has been wiped away to a considerable depth. In such extreme examples it is difficult even to see the Malpighian bodies, but in some cases they are much enlarged and conspicuous, sometimes with an opaque, yellowish, central fleck in each. Ordinarily the splenic pulp in such swollen spleens has a velvety or pasty appearance, and is very opaque and of a dull, pinkish-grey colour.

It must not be imagined that this advanced change is the rule. In a survey of a few hundred cases in the autopsy records of the Johns Hopkins Hospital I found an infinite variation in the degree of gross change. In many instances a note had been made to the effect that the spleen did not seem to be enlarged and had not the appearance of an "acute splenic tumour," but in some of these there were quite definite microscopical changes similar to those seen in the more pronounced cases.

Before describing the microscopical changes in such spleens it will be necessary to say something about the normal histology of the spleen as it was worked out by Weidenreich[2] and Mollier.[3] The Malpighian bodies are collections of lymphoid cells lying in a reticulum formed from the adventitia of arterioles. After leaving the Malpighian bodies the branches of these arterioles empty each into one of the wide venules which make up the bulk of the splenic pulp. The walls of these venules are formed of elongated endothelial cells whose central nucleus is large, and causes a bulging at the middle point of the cell, which projects into the lumen of the venule. Outside these endothelial cells there is a reticulum of elastic fibrils connecting with the general reticulum of the pulp. Weidenreich states that there is also an intervening structureless basement membrane. In the spaces between the venules there lie the cells of the splenic pulp, about which we know least and are, in this paper, most concerned. Many red cells and various mononuclear cells are normally found there; polymorphonuclear leucocytes are present in small numbers. The question as to whether any of the mononuclear cells are peculiar to the spleen or whether they contribute at all to the circulating blood is an open one. There seems to be no doubt that in such conditions as osteosclerotic anæmia, resulting from tumour metastases in the marrow-cavity of long bones, myelocytes can be formed in the splenic pulp, giving it some of the characteristics of bone marrow. It is also certain that some of the mononuclear cells of the pulp are phagocytic; the endothelial cells of the venules and some of the large reticulum cells are often found laden with pigment.

Various authors, such as Evans,[4] Kozumi[5] and Goldzieher,[6] have attempted to classify different types and degrees of splenic change in acute bacterial infections on an elaborate ætiological basis; but the only divisions for which there seems to be any justification are the "red" and "grey" types, the former occurring in typhoid, and the latter in the pyogenic infections which we shall consider.

In a microscopical study of these spleens one can see with the low power (Fig. 1) that there is a great increase in the number of cells in the pulp, so that the sinuses are compressed and, in the more advanced cases, almost empty of blood.

In some of the more extreme cases it is practically impossible to distinguish the outline of the sinus walls, and it looks as if there were an actual solution of the supporting reticulum; this has been described by Goldzieher,[6] who suggests that damage to the reticulum fibrils accounts for the softness and friability of the spleen. But similar damage to the reticulum was seen in some of my experimental animals which died during the night, and in these there were no other alterations in the spleen, while freshly-fixed "acute splenic tumours" did not show such changes; thus it is possible that a large part of the change in the reticulum is due to post-mortem autolysis.

By examination under a higher power one can see that the increased cellularity of the pulp results from the summation of many factors. There is some proliferation of the endothelium lining the sinuses; whether this is a regenerative process following damage to the original endothelium or merely a preparation for increased activity of the spleen it is difficult to say. Phagocytosis is not a prominent feature. Proliferation of the reticulum is not apparent except, perhaps, in cases where there has been a long-standing sub-acute infection. In the pulp between the walls of the sinuses there is a variable number of polymorphonuclear leucocytes, and, most noticeable of all, an infinite variety of mononuclear cells. The Malpighian bodies show almost as great a diversity of

### PLATE X.

Fig. 1.

(Low power.) Spleen from a case of general peritonitis. Note increased cellularity of pulp and a ragged follicle.

Fig. 2.

(Oil immersion.) Same section as Fig. 1. Note large basophil mononuclear cells, b.m.c., and smaller myeloid cells, m.c.

change as does the pulp. In some instances, more particularly in children, they are greatly enlarged, and the "germinal centres" are considerably hypertrophied, often, apparently, at the expense of the surrounding zone of lymphocytes. It is this change in the centre which produces the gross appearance of yellowish central flecks. The cells accountable for this alteration have abundant pale-staining, non-granular cytoplasm and a large round nucleus with rather scanty chromatin and one or two small nucleoli. Such cells are also found towards the periphery of the follicle and occasionally in the adjoining pulp; they have been described by Washkewitz[12] as peculiar to diphtheria, but they are found equally commonly in other infections, especially in young people. Rather more frequently the Malpighian bodies are ragged, without any proliferation of the "germinal centres," and many of the lymphocytes are pyknotic and being phagocytosed by wandering cells; they may even appear like focal necroses.

Examination under oil-immersion enables one to differentiate the mononuclear cells of the pulp in this condition into at least four groups (Fig. 2):—

(*a*) Lymphocytes, similar to those in the follicles and in the blood-stream.

(*b*) Small plasma cells which have the characteristic appearance when stained with the Unna-Pappenheim methyl-green-pyronin stain or with eosin-methylene-blue.

(*c*) Myelocytes, neutrophil and eosinophil, whose granules are well seen with Wright's stain.

(*d*) A large group of cells with a granular but basophil cytoplasm, whose size varies from that of the ordinary myelocytes up to the large cells, which are

P

clearly seen under low power, with a diameter about twice that of a normal myelocyte.

These last cells are described by Huebschmann,[7] Kozumi[5] and others as plasma-cells; they refer to the largest cells as "lymphoblastic" plasma-cells. Huebschmann admits that they do not give the typical staining reaction of plasma-cells, particularly in that there is no peri-nuclear halo; the cells to which he refers are well illustrated in his paper and are identical with the ones we are considering. He mentions the large "lymphoblastic plasma-cells" inside the lymphoid follicles, and remarks that their cytoplasm is paler than that of the cells in the pulp. The nuclei of those cells in the follicles are practically identical with those of the large cells in the pulp; in each case the nuclear material is rather loosely arranged, and there are from one to three nucleoli; there is no suggestion in any of these cells of the typical wheel-spoke arrangement found in plasma-cells.

Goldzieher suggests that these large basophil cells in the pulp may arise from sinus endothelium, because he has observed developing basophilia of the lining endothelium and desquamation of these cells into the sinuses. One is inclined to doubt the truth of this suggestion, because these endothelials are smaller and most of the cells we are considering appear in the pulp between the sinuses.

As early as 1900 Dominici[8] described these large basophil cells in the spleen in acute infections. He noticed that they were of different sizes and thought them lymphoblastic; he differentiated them from true plasma-cells which, he said, were also present but in smaller numbers. In long-continued infections he found they are apparently changed into basophil myelocytes.

Ziegler[9] describes similar cells in the spleen in myeloid leukæmia and considers them myeloblastic. Schridde[10] states that the changes in acute splenic tumour represent a myeloblastic reaction, and sees the same large mononuclear cells in myeloid leukæmia ; he identifies these with the " large plasma-cells " of other authors and believes them to be myeloblasts. Neither of these authors gives any definite reason for his belief. The work reported in this paper represents an attempt to prove or disprove these views.

I find many of the smaller basophil mononuclear cells very like myelocytes, and with a Wright's stain a few fine granules are seen in the cytoplasm. When the larger cells are undergoing mitosis one also finds granules in the cytoplasm.

An oxydase stain of such a spleen shows a considerable increase of oxydase-positive cells. Great difficulty has been experienced in accurately identifying the cells which give this reaction because of the manner in which the granules obscure the nucleus. I found it more satisfactory to stain thin frozen sections with pyronin or safranin and examine them wet, without a cover-slip ; focusing on one cell which could be recognized as one of the large mononuclear cells under discussion and then running on to the slide, still under the microscope, a solution of Winkler's oxydase stain. In this way it was possible to discover which cell reacted. After repeating this procedure on many different cells the conclusion was reached that the oxydase reaction was given by the smaller of the mononuclear cells but not by the very large ones. It was also positive, of course, in the myelocytes, polymorphonuclear leucocytes and in those phagocytic endothelial cells which contained other cell debris. This conclusion agrees with the impression that one gets on examining an ordinary oxydase stain of the spleen in these infections. It is generally accepted that myeloblasts do not give an oxydase reaction; this was worked out by Dunn[13] in a paper on the use of the reaction in the diagnosis of leukæmias. It is equally well known that the more mature cells in the myeloid series, myelocytes and polymorphonuclear leucocytes, do give the reaction; hence these observations led one to think that the whole series might be of myeloblastic origin, particularly as a great similarity is found between many of these cells and the agranular myeloid cells in the bone-marrow.

The microscopical changes described above are those usually encountered in adults. In infants under one year the picture is so different as to require a separate description. There is an increase in size due to increased cellularity of the pulp and, often, increase in the number of cells in the "germinal centres" of the Malpighian bodies, and the gross appearance may not be very different from that of adults, but it is in the nature of the cellular change that the difference lies. There is an increase in the physiological extra-medullary blood formation; one sees numerous myelocytes and some normoblasts. The picture is very similar to that seen in congenital syphilis, where there is an abnormal persistence of the embryonic blood-formation, but in the latter the change is usually rather localized, the normoblasts and myeloid cells appearing in groups in the pulp. It seems that in acute pyogenic infections the cells are more scattered and there are fewer cells of the erythrocyte series. It may be remarked that this state of affairs is found in the absence of any suspicion of congenital syphilis.

The interpretation of these microscopical findings is not easy, as one may judge from the divergent views of many authors. Jawein[11] regarded the swelling of the spleen as a process associated with the destruction of red corpuscles, and found that it occurred only in those intoxications and infections in which there was much blood destruction; he considers the cellular hyperplasia of the pulp to be purely phagocytic. I rarely saw any erythrophagocytosis worth mentioning in my spleens, and do not agree that the majority of the mononuclear cells exercise any phagocytic action at all. In the typhoid spleen this phagocytic explanation undoubtedly holds. Schridde and Ziegler, as already stated, considered the change myeloblastic in origin. More recently Bykowa[14] published some work on animals which demonstrated the appearance of myeloid cells and what he describes as hypertrophied reticulo-endothelial cells in the splenic pulp after injection with toxins and bacteria. He injected scarlet fever toxin into mice, and when the doses were sub-lethal and repeated he found these changes; similarly he produced myelopoiesis by injection with living diphtheria bacilli together with diphtheria toxin. Similar results were obtained with dogs, myeloid cells appearing in the spleen, liver, lymph nodes and adrenals. He does not appear to have kept any record of the leucocyte count during the experiments. Morris,[15] in 1907, induced anæmia in rabbits with pyrodin and found compensatory hyperplasia of the bone marrow and, in the spleen, large non-granular mononuclear cells like those in the bone marrow, varying in size from that of red cells to more than twice that diameter, and having very basophilic cytoplasm and a paler nucleus. From these large cells he found all gradations down to typical granular myelocytes. Neither Bykowa nor Morris applied these observations to the study of the spleen in acute infections, but their accounts of the cells they found tally closely with those described above.

In order to investigate more fully the ætiology of the changes in the spleen it was thought advisable to make a detailed study of a number of cases in the autopsy records of acute infections, irrespective of whether they were said to have "acute splenic tumours" or not. I therefore drew up, in tabular form, lists of 180 cases in which full clinical and pathological data were available. It was found possible after prolonged study of these spleens to make a mental estimate of the degree of splenic change by observing the relative number of large mononuclear cells present in the pulp. It is admitted that this involves a large personal factor, but other methods, such as making differential cell counts, although sounding more scientific, are extremely cumbersome and probably no more accurate.

The data collected in these cases were the age of the patient, the nature of the disease, the duration of the disease, the pathogenic organism, the leucocyte count before death, and notes on the examination of the spleen and bone marrow. The age factor was important because, as has been mentioned, there are certain differences in the form of the reaction to infection in infants and young children. It was found impracticable to formulate any relation between the duration of the disease and the degree of splenic alteration, because of the almost invariable presence of a more active phase of the infection at the termination. The nature of the disease was of no statistical importance, a pneumococcal peritonitis producing the same sort of change as a pneumococcal infection of like virulence elsewhere. The most noteworthy results were obtained by a consideration of the degree of leucocytosis attained before death, and for this purpose it was found advisable to divide the cases into three age groups: those under one year, those between one and five years, and those over five. A summary of the results is given here in tabular form. As one has already mentioned, a mental estimate of the degree of splenic change was made by observing the relative number of large mononuclear cells in the pulp. In these tables "0" means that none of the large cells were present, while one, two, three and four "+" signify an increasing number.

*Under one year old.* 65 *cases.*

| Degree of splenic change .. .. | 0 | + | ++ | +++ | ++++ |
|---|---|---|---|---|---|
| No. of cases .. .. | 36 | 18 | 9 | 2 | None. |
| Average leucocyte count in thousands | 16·8 | 19·4 | 26·8 | 19·5 | — |

*Between one and five years.* 24 *cases.*

| Degree of splenic change .. .. | 0 | + | ++ | +++ | ++++ |
|---|---|---|---|---|---|
| No. of cases .. .. | 4 | 4 | 11 | 5 | None. |
| Average leucocyte count in thousands | 20 | 28 | 25·6 | 26·8 | — |

*Over five years.* 90 *cases.*

| Degree of splenic change .. .. | 0 | + | ++ | +++ | ++++ |
|---|---|---|---|---|---|
| No. of cases .. .. | 8 | 18 | 30 | 32 | 2 |
| Average leucocyte count in thousands | 13 | 14·4 | 20 | 21·5 | 27·5 |

It will be seen from these tables that in infants, while the majority of cases showed no large mononuclear cells in the spleen, those in which they were present had on the whole a higher leucocyte count. In children between the ages of one and five an increasing number had the cells present, and there was a rough agreement between the number of cells and the leucocytosis. In persons over five, however, the results were much more striking. It was found here quite definitely that where there was a high leucocytosis there was also a high degree of splenic change. There were a few rather notable exceptions, which will be discussed later, but even though they were included in these statistics the averages conform with the thesis.

Before one could begin to generalize upon the part played by a demand for leucocytes upon the production of " acute splenic tumour " it was necessary to explore some other possibilities. It was suggested, for instance, that as pyrexia was a factor common to all these infections it was desirable to discover whether an elevated body temperature, in the absence of pyogenic infection, could produce any such changes in the spleen. To investigate this possibility a series of autopsies was collected in which there had been pyrexia as a result of cerebral injury.

Six cases were found where there had been pronounced pyrexia (100°–107°·6) for periods of fourteen hours to seven days, and in none of these was there any change in the spleen. To investigate the question further, pyrexia was produced artificially in rabbits by placing them in an incubator and gradually raising the temperature from that of the room to a maximum of 44° C. The results of these inquiries were that the spleens of six human cases of pyrexia of cerebral origin were found to show no changes similar to acute splenic tumour, and the spleens of five rabbits whose temperatures were artificially raised in an incubator to points corresponding with those obtaining in the course of acute infections showed no changes. The rabbit spleens were compared with those of six normal controls and with those of at least twenty which had acute pyogenic infections and the usual concomitant reaction in the spleen. It is concluded that pyrexia alone is not responsible for the presence of any of the cells found in acute splenic tumour.

There is one other factor which is common to all infections, and that is the production of antibodies, and since little is known about the actual mechanism of antibody formation it was thought necessary to consider the possibility of a relation between the changes in the splenic pulp with this function, especially as it has been abundantly proved that the spleen is an important organ in this respect. In 1898 Pfeiffer and Marx,[16] working with cholera, found more immune bodies in the spleen and bone marrow than in the blood, and also discovered that while splenectomy does not prevent subsequent immunization, it very greatly diminishes it if the operation be performed just after a course of immunization has been begun. At the same time Wassermann[17] and Wassermann[18] found antibodies demonstrable in the spleen, bone marrow and lymph nodes several days before they could be detected in the blood. Van Emden[19] confirmed this. In 1913 Tsurumi and Kohda[20] injected dogs intravenously with typhoid bacilli and found agglutinins earlier in the spleen than elsewhere; they also noted decreased antibody formation in splenectomized animals. In the same year Przygode[21] grew splenic pulp cells in tissue culture and was able to demonstrate the production of precipitins and agglutinins when horse serum and typhoid bacilli respectively were added. Schilf,[22] in 1926, found a vibriolysin produced in tissue cultures from the spleen of rabbits and guinea-pigs by the addition of various killed spirochætes. The baneful effects of splenectomy on antibody formation were further demonstrated by Morris and Bullock,[23] and in 1925 Lauda[24] showed that the so-called pernicious anæmia of rats which occurs after splenectomy was the result of a loss of immunity to a latent infection with an organism of the *Bartonella* group.

The following experiments were therefore carried out with a view to determining whether during the active formation of antibodies, in the absence of infection, there appear in the spleen any of the large mononuclear cells found in acute infections. It was decided to use cats for this work, because the normal cat spleen is more like that of man than almost any other laboratory animal, and also the changes occurring in it in acute infections are quite comparable with those seen in man.

Seven normal healthy cats of different ages were killed for controls, complete autopsies performed, and a study made of the spleens and bone marrow. It was found that there were usually a number of small mononuclear cells along the borders of the trabeculæ in the spleen; these are important, in that when a cat died during the night there was a great proliferation of these cells, and care had to be taken to differentiate them from the cells of "acute splenic tumour"; these cells became phagocytic, and possibly played a part in the solution of the reticulum, which has already been mentioned. (Fig. 3.)

Twenty cats were infected with different pyogenic

### PLATE XI.

Fig. 3.

(High power.) Normal cat spleen.

Fig. 4.

(Low power.) Spleen of a cat infected with staphylococcus aureus. Note cellularity of pulp as in Fig. 1.

Fig. 5.

(High power.) Same section as Fig. 4. Compare with Fig. 3.

### PLATE XII.

Fig. 6.

(Oil immersion.) Same field as Fig. 5. Compare with Fig. 2. Note large basophil mononuclear cells, b.m.c., and smaller myeloid cells, m.c.

Fig. 7.

(High power.) Spleen of a cat treated with staphylococcus aureus vaccine. Compare with Figs. 3 and 5. Note also the large basophil cells, b.m.c., and smaller myeloid cells, m.c.

organisms by various routes, records kept of their daily temperatures and white cell counts made at intervals. At autopsy sections were made of the spleen and bone marrow and of various other organs involved in the infection. Autopsies were also performed on cats which died of intercurrent infections, such as distemper.

It was found that cats are highly resistant to streptococci. Attempts were made to infect them with cultures of *S. viridans* from a human case of ulcerative endocarditis without effect. Infection with pneumococci was also difficult to obtain. Most of the work was done with staphylococci.

Spleens of twenty cats which had infections with staphylococci and pneumococci, and also those of cats dying of distemper and a few other intercurrent infections, were examined and found to show changes in every way similar to those seen in man. The accompanying microphotographs (Figs. 4, 5 and 6) show the typical large mononuclear cells. The bone marrow was hyperplastic wherever the splenic change was noticeable, and both these alterations corresponded roughly with the degree of leucocytosis developed.

A series of five adult cats were then immunized to diphtheria toxin. Blood serum was taken at the beginning and at the end of the experiment.

1. The first cat of this series was given five doses of toxin-antitoxin (0·5 c.c.) followed by 9 m.l.d. of diphtheria toxin spread over nineteen days. At the end there was paralysis of the hind limbs. The white cell count at the beginning was 12,000 and at the end 11,000.

2. The second cat had four doses of toxin-antitoxin and 29 m.l.d. of diphtheria toxin over a period of twenty-one days. Paralyses appeared and the cat was killed. The white cell count at the beginning was 13,000 and at the end 11,500.

3. The third cat had five doses of toxin-antitoxin and 124 m.l.d. of diphtheria toxin over a period of twenty-nine days. The last three doses of toxin consisted of 20 m.l.d. on three successive days. There were no paralyses. The white cell count at the beginning was 14,500 and at the end 13,000.

4. The fourth cat had four doses of toxin-antitoxin only over a period of twelve days. The white cell count at the beginning was 14,000 and at the end 15,000.

5. The fifth cat had five doses of toxin-antitoxin and 114 m.l.d. of diphtheria toxin over a period of twenty-nine days, the last three doses consisting each of 20 m.l.d. The white cell count was 15,000 at the beginning and at the end of the experiment.

The diphtheria toxin was standardized immediately before use.

The sera from these cats were injected into guinea-pigs together with lethal doses of diphtheria toxin in corresponding proportions, due consideration being given to the differences in weight of the animals. The sera which were obtained from the cats before treatment with diphtheria toxin exercised no protection towards the guinea-pigs, and they all died within two days and a half, but the sera collected at the end of the experiment were, with one exception, definitely protective. The exception was that from cat 4, which had toxin-antitoxin only. Guinea-pigs protected with sera from cats 1, 2 and 5 did not die at all, and those which had serum from cat 3 lived many hours longer than the controls. It was therefore concluded that four of the cats had produced considerable quantities of diphtheria antitoxin during the course of the experiment; the fact that they could at the end stand large doses of toxin (20 m.l.d.) on three successive days was almost sufficient proof of this, a single dose being lethal to average-sized control cats.

A microscopical study of the spleens of these cats showed no increase of mononuclear cells and none of the large basophilic cells at all; there was pyknosis of many of the lymphocytes in the follicles in all except cat 4, which was entirely normal. The bone marrow showed no change.

To summarize, then, four cats in whose sera high titres of diphtheria antitoxin were demonstrated after treatment with toxin, showed no changes in the spleen similar to those seen in acute infections.

Vaccines were prepared from cultures of two virulent strains of *Staphylococcus aureus* which had been shown to produce infections in cats and the characteristic changes in the spleen. Five cats were treated with daily doses of these vaccines for periods varying from ten to thirty-two days. Blood-serum was taken at the beginning of the experiment and found to contain no agglutinins. The vaccines were cultured before and after use and found sterile. White cell counts were made at intervals, and in each case a high leucocytosis (from a normal of 13,000–15,000 to 21,000 – 39,000) ensued. Agglutinins could only be demonstrated in low dilutions (1 in 5) of the final serum in two cats; in the other three no agglutinins were found. At autopsy the organs appeared normal, but microscopically there was marked hyperplasia of the bone marrow and a change in the spleen in all respects similar to that seen in acute pyogenic infections. (Fig. 7.) There were numerous large mononuclear cells with basophil cytoplasm, myelocytes and polymorphonuclear leucocytes.

In summary, therefore, five cats treated with *Staphylococcus aureus* vaccines developed a high-grade leucocytosis and barely demonstrable anti-bodies. The spleens were not grossly enlarged or softened, but showed changes identical with those found in acute pyogenic infections of mild virulence.

### DISCUSSION.

A microscopical study has been made of large numbers of spleens from autopsies on persons dying of acute pyogenic infections and the application of micro-chemical tests, such as the oxydase reaction, suggests very strongly that the principal change is one of extra-medullary myelopoiesis ; the large cells which have previously been described variously as plasma cells, endothelial cells, and the like are in reality myeloblasts, similar in function if not in origin, to those of the bone marrow.

An analysis of a number of such cases supports this thesis by showing a close agreement, on the whole, between the degree of leucocytosis achieved before death and the extent of the splenic change. There were, however, some cases which were apparently contradictory, and a discussion of these is essential. One was a man of forty-three who had cystitis and pyelonephritis for a month ; his leucocyte count did not rise above 8,800, and yet he had quite an advanced acute splenic tumour. His general reaction was poor ; the bone marrow was scanty and there were not many myelocytes in the sections. One would be inclined to explain the splenic change here as a last desperate but unsuccessful effort to produce leucocytosis. Another precisely similar case in a man of fifty-four may be explained in like manner. Then there were two cases of extensive general peritonitis after appendicitis where the leucocyte counts were 5,000 and 9,000 respectively, and hyperplasia of the bone marrow and advanced splenic change were present. One may suggest that in these instances the destruction of leucocytes in the peritoneal exudate was so great as to exhaust the supply in the blood-stream.

The condition of so-called agranulocytic angina, in which there is extreme leucopenia in the presence of pyogenic infection, is an interesting one in this connection. In those cases that were found in the Johns Hopkins autopsy records there was, as a rule, a very loose splenic pulp with few cells. There were some myeloid cells present and a very few of the large basophilic mononuclear cells, which I regard as myeloblasts, but practically no polymorphonuclear leucocytes either in the pulp or in the sinuses; in these cases the bone marrow was also poor in cells. Here, then, is a disease in which, were there not some factor, probably toxic, which inhibits leucocytosis, one would certainly find an advanced acute splenic tumour.

There was also an interesting case of typhoid fever in a child of four years in whom there was a terminal infection with a pneumococcus lasting three weeks. During this time the leucocyte count rose from the low value obtaining in typhoid to 24,000/c.mm. The bone marrow was definitely hyperplastic, and the spleen showed quite a well-marked change in which the characteristics of typhoid infection were present, together with those of acute splenic tumour.

In cases of sub-acute rheumatism which died in an attack no changes were found in the spleen, and there was no leucocytosis.

Another point of importance arising from a study of these autopsies is that whenever there was an " acute splenic tumour " there was also hyperplasia of the bone marrow. The same was true in all my experimental animals.

With relation to the question of the association of immune body formation and the condition of acute splenic tumour, I have, in addition to my own experiments, had the opportunity of examining the spleens of rabbits and guinea-pigs which had been recently immunized against pneumococci and other organisms by other persons working at Johns Hopkins. These animals were given smaller doses of antigen than I used, and time was allowed for the formation of easily demonstrable anti-bodies. In these spleens there was no suggestion of any change. No record was made of the blood counts in these cases, but the fact that I found in my animals splenic changes with leucocytosis and without anti-body formation is very significant. It will be remembered that I also produced considerable amounts of diphtheria antitoxin in my cats without leucocytosis or splenic change, and diphtheria, unlike typhoid, is a disease where, in man and animals, leucocytosis and acute splenic tumour are found.

It now remains to decide the origin of these large myeloblastic cells in the spleen, and here one can speak with very little confidence. The same difficulty is experienced in attempting to account for the appearance of myeloid cells in the spleen and elsewhere in such conditions as osteosclerotic anæmia. Ehrlich's original view that the myeloid cells reached other organs from the bone marrow by way of the blood-stream is generally discredited, largely because they are not found in the blood. Jolly and Rossello,[25] in a study of the development of the rat's spleen, found that primitive splenocytes gave rise later to cells with finely granular basophil protoplasm, and these to eosinophil and neutrophil myelocytes, mast cells, etc. They thought also that the lymph cells of Malpighian bodies were derived from primitive splenocytes. Megacaryocytes appeared later, and seemed to have a similar origin. The development of the splenic pulp ran parallel with that of the bone marrow, but there were fewer granular cells in the spleen ; it appeared that myelopoiesis in the spleen was less important than in the bone marrow.

Whatever may be one's views on the origin of blood cells it is certain that myeloid cells are produced in the adult spleen when occasion demands.

### SUMMARY.

1. In acute pyogenic infections splenomegaly is due to an increase in the number of cells in the pulp, and in the Malpighian bodies in some cases.

2. The most striking cells which are present are large basophilic non-granular mononuclear cells which are identical with myeloblasts.

3. The degree of splenic change corresponds with the leucocytosis.

4. The identity of the infecting organism is of less importance *quâ* the spleen than the grade of leucocytosis it produces.

5. Pyrexia alone does not produce " acute splenic tumour."

6. The production of " acute splenic tumour " is not essential for, or directly related with, anti-body formation.

### REFERENCES.

[1] MacCallum, *Text-book of Pathology*, 1925, 528
[2] Weidenreich, *Arch. f. mikr. Anat.*, 1901, lviii. 247.
[3] Mollier, *Arch. f. mikr. Anat.*, 1911, lxxvi. 608.
[4] Evans, F. A., *Johns Hopkins Hosp. Bull.*, 1916, xxvii. 356.
[5] Kozumi, *Verhandl. d. japan. path. Gesellsch.*, Tokyo, 1916, vi. 120.
[6] Goldzieher, *Arch. Path. and Lab. Med.*, 1927, 3, 42.
[7] Huebschmann, *Verhandl. d. deutsch. path. Gesellsch.*, 1913, 16, 10.
[8] Dominici, *Arch. de med. experim. et d'anat. path.*, 1900, 12, 733.
[9] Ziegler, *Fol. Hæmat.*, 1908, 6, 113.
[10] Schridde, *Centralblatt f. allg. Path. u. path. Anat.*, 1908, xix. 865.
[11] Jawein, *Virch. Arch.*, 1900, clxi. 461.
[12] Washkewitz, *Virch. Arch.*, 1900, clix. 137.
[13] Dunn, *Quart. Journ. Med.*, 1913, 6, 293.
[14] Bykowa, *Virch. Archiv.*, 1927, 265, 226.
[15] Morris, R. S., *Johns Hopkins Hosp. Bull.*, 1907, xviii. 200.
[16] Pfeiffer and Marx, *Ztschr. f. Hyg. u. Infektionskr.*, 1898, xxvii. 272.
[17] Wassermann, A., *Berlin. klin. Wochnschr.*, 1898, xxxv. 209.
[18] Wassermann, M., *Deutsche med. Wochnschr.*, 1899, xxv. 141.
[19] Van Emden, *Zeitschr. f. Hyg.*, 1899, xxx. 19.
[20] Tsurumi and Kohda, *Zeitschr. f. Immunitatsf.*, 0, 1913, 19, 519.
[21] Przygode, *Wien. klin. Wochnschr.*, 1913, 26, 841, and *ibid.*, 1914, 27, 201.
[22] Schilf, *Centralblatt f. Bakteriol.*, 1926, i. 97, 219.
[23] Morris and Bullock, *Ann. Surg.*, 1919, 70, 513.
[24] Lauda, *Virch. Arch. f. path. Anat.*, 1925, 258, 529.
[25] Jolly and Rossello, *Compt. rend. Soc. de biol.*, 1909, lxvi. 40.

## Figures and Tables

**Fig. 1. f1:**
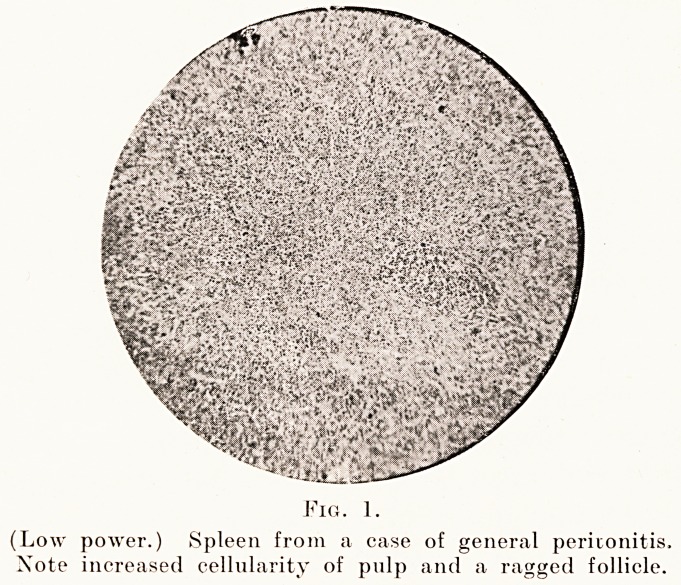


**Fig. 2. f2:**
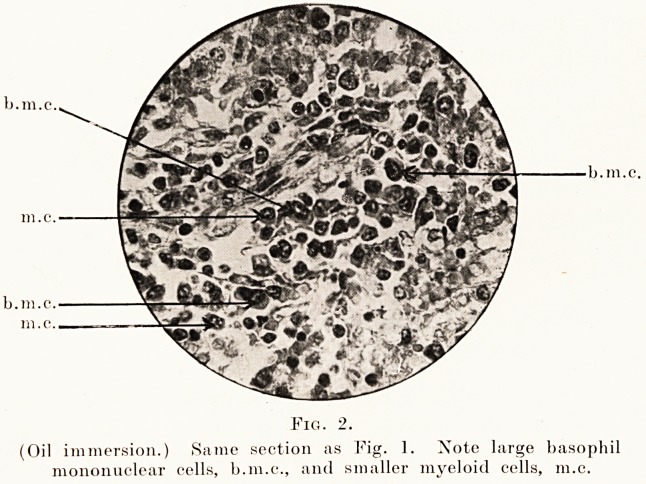


**Fig. 3. f3:**
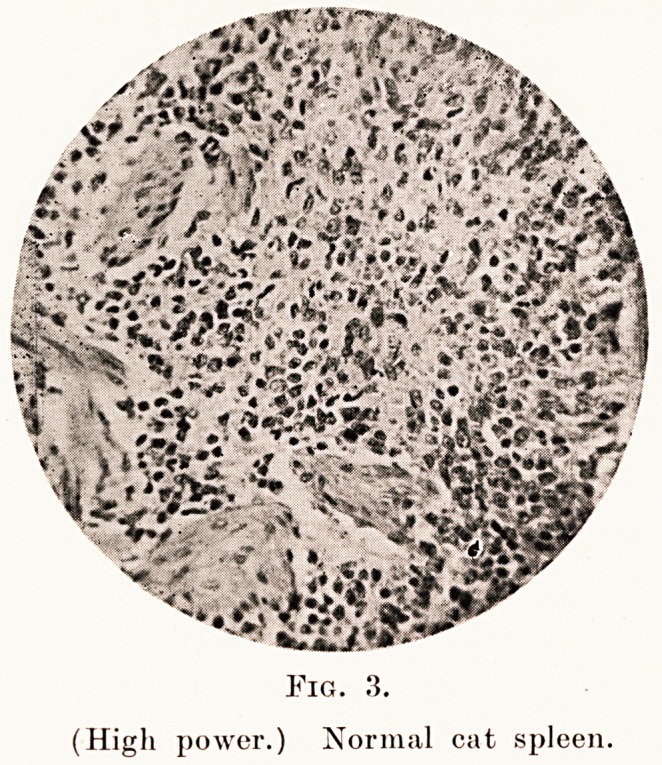


**Fig. 4. f4:**
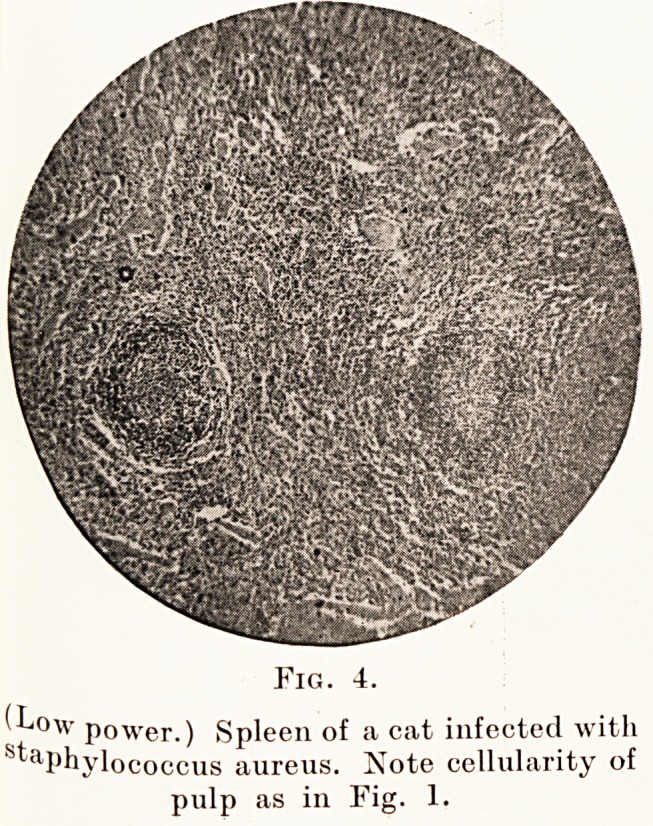


**Fig. 5. f5:**
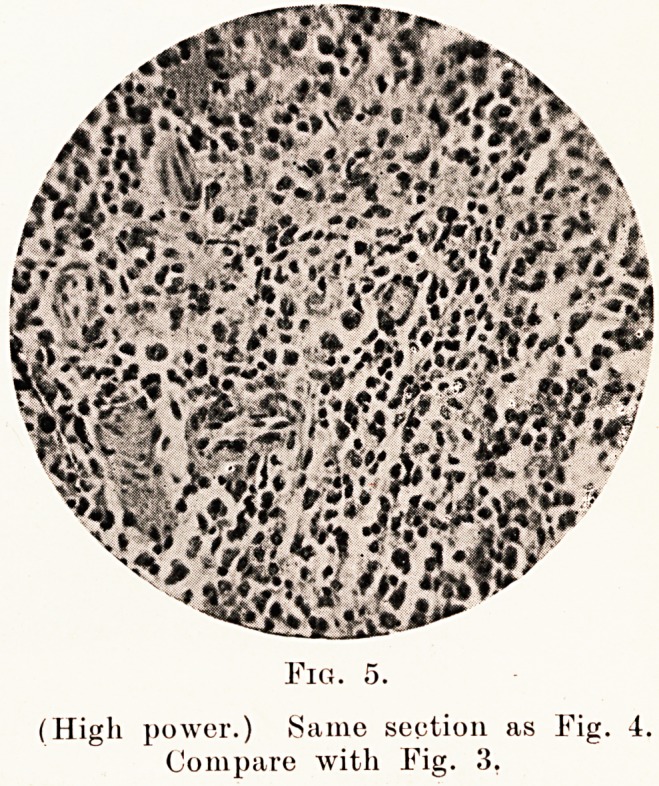


**Fig. 6. f6:**
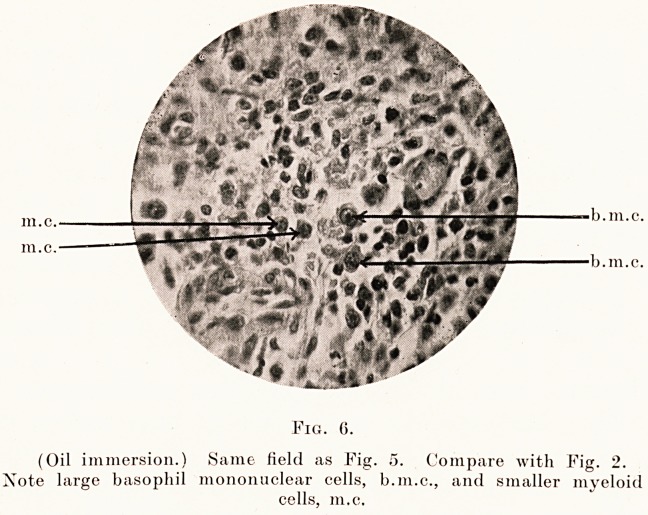


**Fig. 7. f7:**